# Public health round-up

**DOI:** 10.2471/BLT.24.010324

**Published:** 2024-03-01

**The growing cancer burden**

A child undergoing cancer treatment at the Western Ukrainian Specialized Paediatric Medical Centre in Lviv, Ukraine in March 2022. According to the International Agency for Research on Cancer (IARC), the global cancer burden is projected to increase by 77% between 2022 and 2050.

**Figure:**
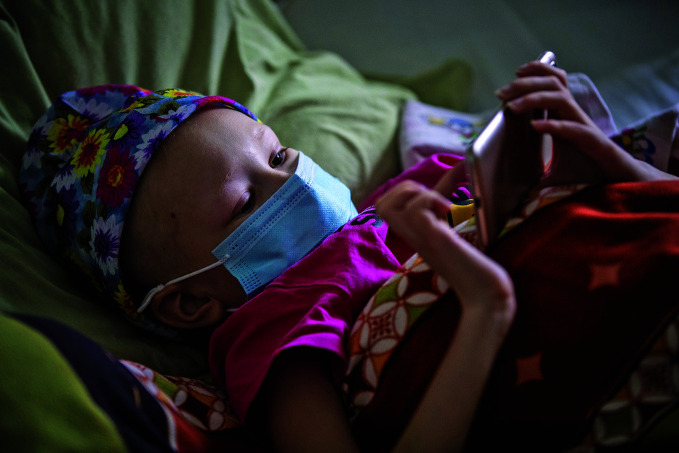
UNICEF/Giovanni Diffidenti

## Sudan health emergency

Sudan’s health emergency intensified, exacerbated by a growing nutritional crisis. In an 8 February statement, the World Health Organization (WHO) reported that nearly 18 million people (37% of the population) are food insecure, with 4.9 million facing emergency levels of food insecurity.

Since the beginning of hostilities in April 2023, an estimated 8 million people have been displaced.

This has led to an increase in the risk of disease outbreaks due to overcrowding and limited access to water, food, and essential health services.

A cholera outbreak initially declared in September 2023 is now impacting 11 of the country’s 18 states, with 10 500 cases reported as of 31 January, and 300 deaths.

Some 70%–80% of health facilities in conflict areas are inaccessible, and disruptions in maternal and child health-care services are further endangering pregnant women and children.

The health cluster, led by WHO, called for financial partners to disburse the entirety of the US$ 178 million funding requested for 2024 to meet the health needs of the highly vulnerable target population.

WHO also urged the international community to advocate for unhindered access to impacted communities and is striving to adapt to the security situation, expanding capacity for cross-border operations to aid partners in inaccessible areas.

https://bit.ly/49cxH93

## Maintaining aid to the Gaza Strip

The allegations of involvement of United Nations Relief and Works Agency (UNRWA) staff in the attacks on Israel that took place on 7 October 2023 prompted several countries to suspend funding, with 13 having done so as of the first week of February.

In a 30 January statement, the Principals of the Inter-Agency Standing Committee (a humanitarian body that operates under the United Nations (UN) composed of both UN and non-UN member organizations) called for a reversal of these decisions, arguing that it was a mistake to prevent an entire organization from delivering on its mandate to serve people in desperate need.

UNWRA has been providing food, shelter and protection to the hundreds of thousands of people left homeless and on the brink of famine in the Gaza Strip as a result of the hostilities between the Israeli Defense Force and Hamas.

“No other entity has the capacity to deliver the scale and breadth of assistance that 2.2 million people in Gaza urgently need,” the principals wrote.

https://bit.ly/3HTVQVX

## Cancer increasing

The global burden of disease imposed by cancer is projected to increase by 77% between 2022 and 2050, rising from an estimated 20 million new cases to 35 million new cases. This is according to WHO’s International Agency for Research on Cancer (IARC) which released its latest cancer burden estimates on 1 February.

The growing global cancer burden reflects both population ageing and growth, as well as changes to people’s exposure to risk factors, several of which are associated with socioeconomic development. Tobacco, alcohol and obesity are key factors behind the increasing incidence of cancer, with air pollution still a key driver of environmental risk factors.

WHO also published survey results from 115 countries, showing a majority of countries do not adequately finance cancer and palliative care services, as part of universal health coverage.

https://bit.ly/3SFU6nY

## Preventing female genital mutilation

This year, nearly 4.4 million girls are at risk of joining the more than 200 million women alive today who have undergone female genital mutilation.

This is according to a statement issued by agencies supporting the Joint Programme on the Elimination of Female Genital Mutilation on 6 February, the International Day of Zero Tolerance for Female Genital Mutilation.

The agencies underlined progress, and noted that in the 31 countries with nationally representative prevalence data, around 1 in 3 girls aged 15 to 19 today are affected. This is down from 1 in 2 in the 1990s.

The agencies called for increased investment in survivor-led movements, as part of efforts to achieve sustainable development goals target 5.3, which includes ending female genital mutilation by 2030.

https://bit.ly/3OHeF2u

## Pooling health technologies

The Health Technology Access Pool (HTAP) will be launched in 2024 to provide a platform for technology partners to voluntarily share intellectual property, knowledge and data in order to accelerate and expand access to technological innovation.

According to a 31 January media release, HTAP will build on the foundation laid by the COVID-19 technology access pool (C-TAP) during the coronavirus disease 2019 (COVID-19) pandemic, while incorporating structural, process and other changes that will enable it to attract and support a range of priority technologies more effectively.

HTAP will focus on pandemic preparedness and response, but at the same time promote access to health products that respond to other public health priorities .

The official launch of HTAP is planned for the second quarter of 2024. In the interim, WHO will adopt the principles and approach described above in evaluating opportunities to secure health technologies and expand regional or global production capacity.

https://bit.ly/3wdyLe5

## Artificial intelligence in health

WHO released new guidance on the ethics and governance of large multi-modal models (LMMs) – a type of generative artificial intelligence (AI) technology with applications across health care.

LMMs can accept a range of inputs, such as text, videos, and images, and can generate similarly diverse outputs. LMMs can mimic human communication and carry out tasks they were not explicitly programmed to perform.

The guidance outlines over 40 recommendations for consideration by governments, technology companies and health-care providers to ensure the appropriate use of LMMs to promote and protect the health of populations.

“Generative AI technologies have the potential to improve health care but only if those who develop, regulate, and use these technologies identify and fully account for the associated risks,” said Dr Jeremy Farrar, WHO Chief Scientist.

https://bit.ly/42AwuWD

**Cover photo**

A child with pneumonia receives care in a rural health clinic in Mankayan, Benguet, Thailand

**Figure:**
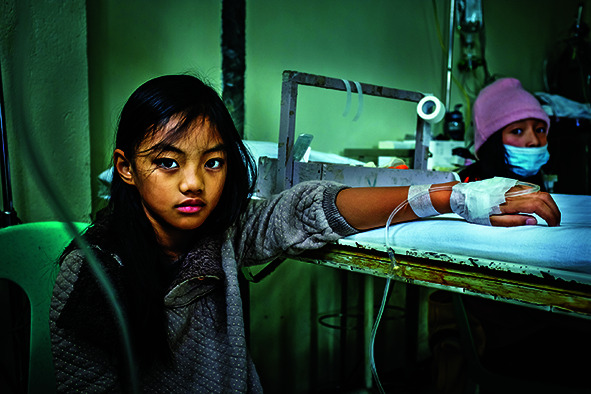
WHO/Yoshi Shimizu

## Action on tobacco and the environment

The Tenth session of the Conference of the Parties (COP10) to the WHO Framework Convention on Tobacco Control (WHO FCTC) concluded on 10 February with a decision to strengthen Article 18 of the WHO FCTC, which focuses on the protection of the environment and people’s health against the harms linked to tobacco cultivation, production and consumption.

In a 10 February statement, Dr Adriana Blanco Marquizo, head of the WHO FCTC Secretariat, said that the decision urges Parties to take account of the environmental impacts from the cultivation, manufacture, consumption and disposal of tobacco products, and to strengthen the implementation of Article 18.

COP10 also strengthened guidelines preventing cross-border tobacco promotion.

https://bit.ly/49vei2O

## Eliminating trans-fatty acids

WHO released results from the first five years of its REPLACE initiative to eliminate industrially produced trans-fatty acids (iTFA). While the ambitious target set by WHO in 2018 –to fully eliminate iTFA from the global food supply by the end of 2023 – was not met, there has been significant progress, including a marked increase in countries implementing best-practice policies for reducing iTFA levels in food.

Fifty-three countries now have policies for reducing iTFA in food, significantly improving the food environment for 3.7 billion people, or 46% of the world’s population, as compared to 6% just 5 years ago. These policies are expected to save approximately 183 000 lives a year.

WHO also awarded its first-ever certificates validating progress in eliminating industrially produced trans-fatty acids to Denmark, Lithuania, Poland, Saudi Arabia and Thailand.

Trans-fatty acids are semisolid to solid fats that occur in two forms: industrially produced and naturally occurring. Intake of TFA is associated with increased risk of heart attacks and death from heart disease.

https://bit.ly/48fJphC

**Looking ahead**

4–5 March. ATACH Global Meeting: Transforming health systems in the face of climate change. Madrid, Spain. https://bit.ly/3OIIuPU

14 March. First high-level meeting to defeat meningitis. Institut Pasteur, Paris, France. https://bit.ly/3SVKy9J

17–18 April. Sixth Global Ministerial Summit on Patient Safety 2024. Santiago, Chile. https://bit.ly/3HZ8YsY

