# Corrigendum

**DOI:** 10.2471/BLT.24.110424

**Published:** 2024-04-01

In: Public health round-up. Bull World Health Organ. 2024, Mar 1;102(3):152–153, 

on page 153, the caption of the cover photo should read “A child patient with pneumonia in a rural health clinic in Mankayan, Benguet, the Philippines.”

